# Exploring and Developing the Questions Used to Measure the Human–Dog Bond: New and Existing Themes

**DOI:** 10.3390/ani12070805

**Published:** 2022-03-22

**Authors:** Lauren E. Samet, Helen Vaterlaws-Whiteside, Naomi D. Harvey, Melissa M. Upjohn, Rachel A. Casey

**Affiliations:** Canine Behaviour & Research Department, Dogs Trust, London EC1V 7RQ, UK; hmvaterlaws-whiteside@outlook.com (H.V.-W.); naomi.harvey@dogstrust.org.uk (N.D.H.); melissa.upjohn@dogstrust.org.uk (M.M.U.); rachel.casey@dogstrust.org.uk (R.A.C.)

**Keywords:** human–animal interaction, dog, bond, questionnaire, dog investment

## Abstract

**Simple Summary:**

Human–animal interactions consist of many relationship types. The human–dog bond is one such example. This study reviewed how we measure the human–dog bond through questionnaires and found a lack of questions related to the dog’s investment in said bond. To rectify this, twelve semi-structured interviews were carried out with a variety of dog guardians to investigate their views on how their dogs showed that they shared a bond with them. The common themes that emerged included ‘affirmation’, ‘understanding of a dog’s preferences, likes, and dislikes’, and ‘adaptation’. These themes provide a useful foundation from which to design new questions within human–dog bond questionnaires. This would allow better representation of a dog’s investment in the bond and, therefore, help create tools that reflect the reciprocal nature of this relationship.

**Abstract:**

Dogs play an important role in many western societies, providing companionship, emotional support, and assistance, as well as other more specialist roles. The literature reveals that many human–animal interaction (HAI) questionnaires exist to measure the human–dog bond (HDB). The first part of this study assessed how far existing questionnaires went in measuring HDB (defined as the unique, dynamic and reciprocated relationship between a person and dog, one in which each member can influence the other’s psychological and physiological state). A systematic literature review revealed that a common limitation in HDB questionnaires was a lack of questions based on the dog’s investment in the bond and, therefore, a failure to measure the two-way characteristic of the HDB. This led to the second part of the study: to identify novel themes relating to dog investment in the HDB from which new tool questions could be developed. This was investigated qualitatively using twelve semi-structured interviews on HDB, undertaken with participants from a variety of dog–guardian relationship types. HDB themes that emerged included ‘adaptation’, ‘understanding of a dog’s preferences, likes, and dislikes’, and ‘affirmation’. Subthemes included ‘boundaries’ and ‘expectations’ (within adaptation), ‘excitement’, ‘proximity’, ‘affection’, and ‘recall’ (within affirmation). The themes that arose provide a foundation from which to build new lines of questioning within HDB tools. Such questioning can better represent a dog’s investment in the HDB and, therefore, help create tools that reflect the reciprocal nature of a bond more accurately.

## 1. Introduction

Within human–animal interaction (HAI) research, the concept of bond formation is common, particularly in discussions of the human–dog bond (HDB). For over 15,000 years, dogs have been co-evolving to meet our needs as “man’s best friend” [1] and the meaning of our relationships with them has continued to evolve too. To understand the human–dog bond (HDB), it is first important to understand what a bond is. In this paper, we define a human–animal bond (HAB), specifically the HDB, as “a unique, dynamic and two-way (reciprocated) relationship between a person and an animal, one in which each member can influence the other’s psychological and physiological state”. This definition was created by amalgamating the most suitable characteristics of several HAB definitions (see Table 1) and by removing any similarities or overlap with the definition for attachment (a lasting psychological connectedness [2]). The key difference between “bond” and “attachment” is that attachment does not need to be reciprocated between the two parties. Table 1 demonstrates the occasionally contradictory nature of existing HAB definitions.

This confusion between HAI and HAB does not end at definitions. The interchangeable use of HAI terms and meanings has led to the design of HAI questionnaires, referred to throughout as tools, which may not always meet their intended purpose (construct validity) [8]. Although such tools are human-centric by nature of their intended users (humans), for those intending to measure the HDB, there is often a notable lack of question representation to assess the dog’s side of the relationship [9,10]. By not assessing the dog’s investment into a bond, tools will only assess the human attachment rather than the bi-directional bond.

Within HAI research, much has been written on the benefits of human guardianship and interaction with companion animals, whether through companionship, emotional support, health benefits, or assistance with tasks [11,12]. While not unreasonable to hypothesise that those animal species that humans form bonds with may feel the same way, we must avoid assumptions without objective evidence. In addition, the wellbeing of dogs within either companion or working relationships needs to be assessed as part of bond evaluation.

Many researchers have developed tools to help assess the value, strength, and benefit of human–dog relationships (for examples, see Table S1 in the Supplementary Materials). While advantageous in being quick and easy to use to assess relationships, the disadvantage of such tools is that they can only ask one party: the human in the relationship. The unequal representation of both parties’ investment in the bond can leave assessments vulnerable to anthropocentric bias.

The first aim of this study was to assess how far existing HAI tools go in measuring an HDB and, therefore, how well they attempt to represent the dog’s side of the relationship. These first investigations highlighted limited evaluation of the HDB based on the dog’s investment in the bond. Thus, the second aim of the study was to identify novel themes based on dog investment in the HDB from which new HDB questions could be developed within HAI tools. This second aim was achieved through use of qualitative interviews with a variety of dog guardians. Due to the dual nature of this study, this manuscript is presented in two parts.

## 2. Materials and Methods

### 2.1. Review of Current HAI and HDB Assessment Tool Questions (Part I)

Based upon the results of systematic literature reviews by Samet et al. [13] and Wilson and Netting [8], 569 HAI questions from 170 tools were identified (some of these questions and tools are referenced in Supplementary Materials, Table S1, the rest can be found in Samet et al. [13] and Wilson and Netting [8]). Using the suitability criteria listed in Table 2, questions which were suitable for use in assessing the HDB were agreed (passed) independently by two researchers (LS and HVW). Any questions with contrasting answers were discussed. “Pass”, “Unsure”, or “Reject” labels were used to determine question suitability; pass/unsure was passed, unsure/reject was rejected, while unsure/unsure or pass/reject results were reviewed and discussed again by both researchers and a final decision was agreed upon. These final questions were then independently categorised for content by the two researchers and grouped into broad themes.

### 2.2. Identifying Novel HDB Themes for Future HDB Questionnaires (Part II)

The themes identified in Part I of the study led to further investigation of novel HDB question themes in Part II via the use of semi-structured interviews with a range of dog guardians representing various human–dog relationship types.

#### 2.2.1. Participant Recruitment

Participants were recruited via convenience sampling of professional and personal networks of the researcher (LS). Although sampling was convenience based, interviewees were recruited based upon their different HDB relationship circumstances to allow for diversity in representation of HDBs and to avoid bias in views (Table 3 provides a list of the types of relationships explored). All interviewees were aged 18 years or over and written informed consent was obtained from all participants prior to interviews. Consent forms highlighted that the interviews were voluntary, not incentivized, and participants could withdraw from the interview and data collection process at any time.

The study aimed to provide preliminary data for a larger project and, therefore, whilst every HDB can be unique and worth investigating, as a time-limited qualitative study, a purposive sample was sought rather than random sampling. Data were collected until saturation was reached (i.e., in line with accepted qualitative research approaches (e.g., [14]), interviews were conducted until similar themes were identified multiple times between interviewees and additional sampling was unlikely to lead to new information being collected). This equated to 12 semi-structured interviews with dog owners or guardians, both of which are referred to as guardians from here on.

#### 2.2.2. Interview Process

The semi structured interviews were based around a written interview guide of predetermined but open-ended questions (Supplementary Materials, Table S2) to allow for broad topics of interest to be covered in question responses, while enabling participants the freedom to articulate their experiences in their own terms [15]. Participants were provided with an overview of question content and the written HDB definition before the interview began, which was repeated again verbally at the start of the interview. The semi-structured interview guide was piloted with eligible Dogs Trust staff (who were not participating with interviews) to refine content before use. Interviews were conducted either in person or over the telephone, and audio was recorded with participant consent before digitising by the researcher (LS). Pseudonyms were created for each participant and any dog/s or family members that were discussed.

Twelve dog guardians were interviewed of ages ranging between 25 and 65. Female guardians were over-represented (75%), as is common in HAI research [16]. Interviews lasted between 20 and 90 minutes with a mean duration of 55 minutes. Relationships between dog and interviewee included companion, working, assistance, social facilitator, breeder, and temporary guardian. Length of relationships varied but all had been established for over 12 months.

#### 2.2.3. Thematic Analysis

NVivo (v.12, QSR International, Melbourne, Australia) was used for inductive thematic analysis of interview transcripts. Themes were then inductively identified from semantic and latent codes by LS [14]. Through systematic data coding, the key themes around guardians’ perceptions of dog investment into the HDB emerged.

#### 2.2.4. Ethical Approval

Ethical approval for this study was granted by the Dogs Trust Ethical Review Board (Reference Number: ERB023). Informed consent was obtained from all subjects involved in the study.

## 3. Results

### 3.1. Review of Current HAI and HDB Assessment Tool Questions (Part I)

From the 569 HAI questions, the researchers independently agreed that 151 questions were suitable for HDB assessment. A further 77 questions were agreed as suitable following discussion (42 initially rated as unsure/pass, 31 as unsure/unsure, and four as pass/reject). Both researchers independently rejected 321 questions, with a further 20 questions being rejected after discussion (18 initially rated as reject/unsure and two as unsure/unsure). This left 228 HDB questions to be independently categorised for content by the two researchers (the 228 questions can be seen in Supplementary Materials, Table S1; meanwhile, Table 4 shows each researcher’s content categorization results). Question content categories formed ten broad themes (Table 5). Only one of the ten HDB question themes was exclusively dog-centred (labelled “dog investment” in the HDB, i.e., emotional investment/attachment), which equated to 18 questions in total (8% of all HDB questions). This indicated that dogs’ perspective of the bond was underrepresented in many current HDB tools. Part II of this study was conducted to begin exploration of dogs’ investment in an HDB in the hope of expanding future question representation from the dog’s point of view.

### 3.2. Identifying Novel HDB Themes for Future HDB Questionnaires (Part II)

Within the semi-structured interviews, three themes emerged from the analysis. The first theme was related to humans’ perceptions of HDB affirmation from their dogs and included the subthemes excitement, proximity, affection, and recall. The second theme was related to an understanding of the dog’s unique preferences, likes, and dislikes (this related somewhat to owners’ attitudes towards whether dogs have, or should have, agency, and whether a guardian had respect for that; this theme often related to the human’s understanding and accommodation of their dog’s emotional needs). The third theme considered the adaptation of both dog and human to that specific HDB, and included the subthemes of a relationship boundaries and expectations from both parties.

#### 3.2.1. Affirmation of HDB from the Dog (Theme 1)

A common theme to emerge from interviews when guardians where asked how they think their dog showed they felt bonded to them was related to some of the responses dogs had to humans. Whether they involved greeting a guardian (excitement), coming when called (recall), choosing to position themselves closely to humans (proximity), and/or affection with said guardian, these responses evoked emotion in guardians (guardians tending to feel positive emotions when related to dogs’ expression of a bond presence, but negative emotions when the opposite was true). From the dog’s perspective, however, many factors other than investment in a bond could play a role in their behaviours described by guardians as affirming; for example, prior training, training methods used, emotional state, temperament, physical comfort, and learnt associations with each behaviour or stimulus. Affirmation was recognized here as a theme because it came up so often in interviews; however, care must be taken to address whether this theme relates directly to a dog’s investment in the HDB in future research.

##### Excitement (Theme 1 Subtheme)

Several interviewees cited their dogs’ showing excitement at reuniting with them after time apart, feeling this suggested a dog had missed them or was pleased to see them as opposed to emotional neutrality which may be assumed with a stranger. This participant describes a time when they felt or feel their bond is strongest with their dog:

“*When I come home from work, he gets what we call the crazies and basically that means it’s just a crazy five-minute period where he’s just like “mum’s home! I’m so excited!” he’s just running around the house, then we have a lil’ tug of war and everything. And I’m just like… it just makes me so happy!*”

(Guardian of a newly adopted dog)

“*He was still massively bonded to his previous owner. His previous owner came to visit us about 18 months after he came to us, just kind of to say, “hi and how’s he doing?” and [Dog] went absolutely berserk when he arrived! He was so excited! I mean, literally did laps, did zoomies [sic], around the garden, you know, he was just so excited to see him!*”

(Current dog’s owner describing the dog being reunited with their old owner)

“*He is very over the top. Yeah, no you almost have to kind of actively discourage the over enthusiasm when he walks in or when you get home.*”

(Guardian in a single-dog household)

This type of behaviour was also mentioned in relation to dogs reuniting with other familiar dogs:

“*We always have a whole re-greeting dynamic even if one’s just been away for 10 min.*”

(Guardian to a multiple-dog household)

Guardians tended to assess the absence or opposite of excited behaviour, i.e., a lack of excitement when being reunited, as not indicating a bond, or there being a possible problem with the relationship. Here, a guardian describes their concern when they initially rehomed a dog:

“*So, at first when we were at home with him, he would sit in his crate, he didn’t really care about being near to us, and so that was part of the reason I thought maybe he’s doesn’t like [us], maybe he’s not happy?*”

(Guardian of a newly adopted dog)

This quote also indicates how the owner felt it was personal whether the dog chose to spend time with them, and how a dog opting to spend time with them was an important signal that the dog liked them and enjoyed spending time with them. The same guardian commented:

“*One evening he just jumped up on the couch and sat between us and we were like ‘Woah, he’s done it! he’s done it—he’s happy!’*”

(Guardian in a single-dog household)

##### Proximity (Theme 1 Subtheme)

Proximity or choosing closeness with a guardian also featured in comments about relationship type:

“*Some of them have been very tactile, where the dog has come to me for lots of fuss. And I’ve also been able to, like, they trust me to touch them pretty much anywhere… …whereas some of them have been more relationships of, for instance, just being nearby, so it’s more of just a companionship rather than a full-on physical relationship.*”

(Guardian of multiple dogs)

While proximity seems important to some dog guardians’ affirmation of the bond, some dogs may have more complicated feelings around human company. Even if a bond is shared between the human and animal, an understanding of the need for space may be favourable to a dog’s social preferences and wellbeing, which may determine how favourably they see their relationship with that human:

“*She [the dog] was quite standoffish in her behaviour, in that she didn’t seek physical contact with you very, very often, or not to start with anyway, she does more now, but at the start she was very self-contained and did her own thing, and would actively at times, she’d sit with you in the room for a little while.*”

(Guardian of an assistance dog)

“*He’ll go and lie by himself up the stairs or at the end of the couch beside you… … He would sometimes come up sit with me, but other times go and sit by himself.*”

(Guardian of an older dog in a multi-dog household)

This understanding of agency could aid empathy towards dogs as individuals with a choice in being part of an HDB. These themes crop up again in the Understanding of Dogs’ Preferences, Likes, and Dislikes section below.

##### Affection (Theme 1 Subtheme)

Many dog guardians appeared to enjoy the affection and tactile closeness that they had with their dogs, indicating that they believed their dog enjoyed this aspect of their relationship too:

“*I know everyone says dogs hate hugs, which is rubbish when it comes to [dog’s name], because [dog’s name] will wrap his arms and legs around you... … [dog’s name] sort of pulls you into him.*”

(Guardian discussing the differences between the dogs in their household)

“*…and in the mornings, he likes to loll[sic] on me, he likes a cuddle.*”

(Guardian describing their daily routine with their dog)

Dogs that enjoy affection may also enjoy it in different ways depending on the relationship they have with the provider/recipient. Here, a guardian describes the difference his and his dog’s bond makes to the dog’s tolerance of affection from other people:

“*Some dogs enjoy that tactile contact, and when it’s a new person she’ll enjoy it with you for a few moments, whereas I could probably go on [with tactile contact] for minutes and minutes at a time with her, with her cuddling with me on the sofa and [me] stroking her.*”

(Guardian in a multiple dog household)

Meanwhile, this guardian describes sharing different affectional bonds with each of their five dogs:

“*I think I am more snuggly with my boys, and they are more snuggly with me, than the bitches are.*”

(Guardian in a multiple dog household)

For some dogs, as with proximity, exchanges of affection are on their terms and not the guardian’s:

“*I think with [dog’s name] it’s probably kind of late in the evening when she’s got into a comfortable spot. If she’s close to you, and she’s kind of in a spot where she will request, you know, that you stroke her head in a certain way, and be very specific about what she wants and she literally will just kind of cuddle in, and just relax completely, and just as long as you keep doing what she wants you to do then she’s totally happy, and that’s lovely from my point of view in terms of going, you know, feel like she’s relaxing and content where she is, and knowing she’s comfortable.*”

(Guardian of a rehomed dog)

##### Recall (Theme 1 Subtheme)

Recall came up surprisingly often in discussion of dogs’ behaviours within the HDB. Poor recall appeared to be related to a guardian feeling frustrated about their dog’s ‘defiance’ of a command that they believed the dog knew the meaning of. Here, a guardian was asked whether there was anything that their dog could do, or that could happen, that might affect their HDB:

“*When she eats cat poo! ..... I don’t like her very much (laughs). No, I don’t think so. I think, her recall’s really bad, so when she doesn’t come back, that makes me more frustrated rather than worried because she just acting like an idiot.*”

(Guardian of an older dog)

“*I would say he’s one of the most difficult cockers I’ve ever had to date, or worked with to date, and I would say that includes other people’s cockers. One minute he’s there and then the next he’s out and off! Hence the reason he has his own hashtag!*”

(Guardian of a multiple-dog household, who also breeds dogs)

This guardian was asked to describe a time they felt the bond had been broken due to poor recall:

“*When walking the dog and a herd of runners ran by and that frightened the dog and the dog panicked and ran off. She behaved in a manner unbecoming of her you know, she freaked out and it took me a while to recover her, for her to come back to me, so I’d lost, I lost the bond in that instance.*”

(Male guardian of a single dog)

Conversely, good recall whilst out together appeared to be associated with the theme of proximity, good behaviour, and of a dog choosing to interact with their guardian when in an outside environment, whether via preference or obedience in recall. These guardians described a characteristic of the relationship they share with their dog when on walks:

“*Walks he always wanted to be near you, around you, he would always come back from other dogs.*”

(Guardian of a single dog)

Interviewer: “*And when he’s out, does he retain a bond with you, or does he have any behaviours that indicate that?*”

Participant: “*He always loops, I mean they go far and wide pointers, they just disappear into the undergrowth and out of sight but [Dog] does specifically loop round every few minutes to check in. He’s not the pointer that kind of disappears over the horizon completely. So, he literally does come [back] and he will literally come running back to you with eye contact kind of like going “I’m here, I’m here. Are you okay? Ok right, I’m off again.” “Yeah he will look directly at you.*”

(Guardian of a multiple-dog household)

“*Yeah. I actually learned a lot from him because he was very much the dog, you know, if you were out walking and you had him off lead, I’d call to him he’d come straight back to me, I’d pop him on the lead and someone’s off lead dog would be coming bounding up and I’d be going “Please put your dog on a lead!” and the person’s going “but he’s friendly” and I’d be going “but mine isn’t!*”

(Guardian of a multiple-dog household)

Obeying a recall command was seen as good behaviour by guardians. A well-behaved dog appeared to be satisfying or pleasing for the owner who trained them. This owner describes their expectation of their dog to be well behaved but also cites factors that may influence that (e.g., age, play):

“*In return, I expect him to, yeah, I mean, be fairly well behaved, umm, which but that’s through training and obviously I can’t expect him to be well behaved without him being trained but I mean he does all that anyway, he’s still young and he likes to play but he is generally well behaved—very well behaved actually I’d say. And when he’s out, he’s no trouble at all, he comes back when he’s called, he’s a pleasure to have as a dog actually.*”

(Co-guardian to a single dog)

When this guardian was asked which of their dog’s behaviours showed them that their dog was bonded to them, they responded:

“*Well, I don’t think it is any one behaviour. I like it when he comes when I tell him to, and he likes to play with me, and I like to play with him, and I can see he’s happy because he wags his tail, and he’s happiest of all when I give him food, so it’s all aspects really, it’s his demur [sic].*”

(Co-guardian to a single dog)

Poor recall out on walks may have had an additional element of frustration, or negative emotional impact to guardians, because of the public nature of the “disobedience”. Judgement by other dog walkers appeared to hold value to some participants, as this guardian explained:

“*I think the thing I don’t like, or don’t like the idea of, is if people think I beat him [in response to the dog not coming back when called]. I’d hate to go into a park, and I call him, and he sort of like puts his head down for some reason, which he could do, and people think “oh look at that poor dog.*”

(Male guardian of a single dog)

Another participant’s thoughts corresponded to this sentiment when discussing the need for good recall from their dogs when out and about:

“*I’m not horrible to my dogs, but you must have, for their safety, you need certain boundaries or certain stringent things that are in place because their safety is important. So, if it means that I’m going to shout, somebody might interpret that as “oh you’re being a bit mean”, but I don’t want that dog going in somewhere unsafe.*”

(Guardian of multiple dogs)

#### 3.2.2. Understanding of Dogs’ Preferences, Likes, and Dislikes (Theme 2)

An understanding of a dog’s greatest positive reinforcers and motivators for training, happiness and wellbeing, commonly featured within the semi-structured interviews. It was particularly important for successful training and a positive working relationship between dog and guardian, where there was a strong functional aspect to the bond.

“*The two boys, I think they get as much out of physical contact and being with me as I do, and [Dog] is a classic example in that he will work much more for physical contact and praise than treats, and I train with treats, I use treats, BUT he’s definitely not a foodie orientated dog. No, he loves you—basically just give him a big physical fuss.*”

(Guardian to multiple dogs)

However, for some dogs, their greatest reinforcers or preferences may be unrelated to anything a single human relationship can uniquely provide them, as this interviewee commented:

“*Sometimes you can get dogs where they’re so they can provide reinforcement to themselves from the environment, and from so many other things, that potentially they don’t pay us [humans] [any attention] in [that] they are not too in tune with the owner, or the owner doesn’t necessarily play a really pivotal role in [their] development, or welfare. Do you see what I mean? Like they [guardians] feed them, and the dog knows that, but they [the dog] [would] also potentially just as happily go off with somebody else. It’s not necessarily a really unique bond in that sense.*”

(Dog guardian who also works with dogs)

Knowing what could scare a dog or when the dog might be uncomfortable could allow guardians to offer support and advocate for their dog when needed:

“*I remember one dog would scare her a lot so my bond would become more supportive. An example might be thunder or fireworks when then I became another role of protector.*”

(Guardian of an assistance dog)

“*We didn’t live right next to the main road, but it was just off our road and up another one and it was there. And it was connecting between two cities, so it was quite busy and a lot of like farm trucks and stuff going past, and I was like we’ll have to do lots of desensitisation and counter conditioning to noisy trucks—didn’t bat an eyelid and there’s lots of buses as well. Doesn’t like cars splashing through puddles though; startles at the noise, of like any sort of noise like the splash of a puddle, or when a bird suddenly takes off from a bush and it’s that kind of like “spshhhh” noise, but not bangs and not claps. It is very specific, like, a specific kind of “spshhh” kind of noise.*”

(Guardian of a rehomed dog)

Knowledge of a dog’s preferences may also lead to other forms of supportive behaviour changes from guardians or encourage guardians to change their behaviours according to the dog’s preferences.

“*He had been an outdoor [dog] when we got him, we realised quite quickly that he preferred to have access to the garden as much as possible. And we did, we actually gave him a kennel in a reasonably sized garden, so we actually had a kennel in the garden and we just basically kind of let him move around.*”

(Guardian of a rehomed older dog)

“*I think one of the reasons I don’t particularly like staying away from home is because lots of hotels and Airbnb’s are really strict about not having the dog on the bed. Which she [the dog] doesn’t like and I really look forward to getting home and having a cuddle with [Dog] in the morning [on the bed] with a cup of tea.*”

(Guardian of an assistance dog)

Lack of understanding about a dog’s behaviour, especially an unwanted one, can highlight to guardians an element of the relationship with their dog that they do not have control of or cannot explain. The size of this element, and the issue it causes for the owner, can place challenges on the bond. Guardians may face the challenge of being able to support their dog with behaviours that are routed in the dog being uncomfortable, upset, or lacking in something needed for good welfare. This could create a characteristic of that bond.

“*You know he’s always been like that and that’s about the only thing about him. I think, why do you do that? and I don’t know. And I don’t think I’ll ever know coz he can’t tell me.*”

(Guardian of an assistance dog)

“*[Dog 1] has had her own different tricky areas, which has taught me so many things, but it’s not taught me to decipher them, just like, “oh, how do I cope with this? So, what do I need to do to work with that”, you know, if I brought [Dog 2] into a new house she’d settle and lie down quite easily. [Dog 1] would to an extent, but I know I could leave [Dog 2], whereas [Dog 1] would be like, “I can’t be left”. So, there is certain scenarios that [Dog 1] needs a bit more work on, she’s reliant on me because she doesn’t like being left.*”

(Guardian of an assistance dog)

Accrediting a dog with agency seemed to support dog guardians’ understanding of their dog and empathy for their feelings and different responses in certain situations:

“*It’s a bit like a mum-child relationship, she definitely gets angry with me, well not angry just stroppy, but at the same time if she’s feeling poorly, or wants something, I’m the first person she comes to.*”

(Guardian of a single dog)

The same guardian remarked:

“*I think [Dog] gets angry with me sometimes—oh she does get cross! She definitely gets cross. If we go somewhere, she doesn’t want to be or something, she will absolutely tell me and she’ll strop about it afterwards.*”

An acceptance and an understanding of these preferences suggested an owner had a respect for that agency. One participant stated:

“*I think she would be very specific about who she engages with and she doesn’t engage with so she would decide whether she wants to be with you or attached to you. And you’ve got to, well we’ve learnt, or we have figured out that you have to kind of respect her choices or ways and just let her, let her kind of direct things to make her comfortable. So, she could be anybody’s as long as they were prepared to respect that. I don’t think she could cope with a house full of children for example, no way. And as we know that she’s worried about men she actively avoids them. Now, with my husband, she’s as cuddly with him as she is with me but that’s taken five years to kind of learn.*”

(Female owner of an older rehomed dog)

However, once again, participant’s answers showed an acknowledgement of other people’s views in relation to this:

“*I am conscious people have said to me “oh you shouldn’t allow that” and I’m like “why? that’s their dynamic”. Now I don’t believe they’re a pack. There is definitely a movable dynamic in our house as to, you know, who goes where, who’s allowed to sleep where, who has what toy and that kind of thing, and I allow them that*”

(Female owner of multiple dogs)

Consistency in a guardian’s preferences appeared to help dogs understand their human’s likes and dislikes, and how to respond to those likes and dislikes:

“*I think what dogs like is rules and they want to know what they are allowed to do and what they’re not allowed to do.*”

(Guardian of an assistance dog)

“*I think he expects sometimes, he will expect me to tell him off. Like if he picks up my shoe—and he likes to show off right—If he picks up my shoe, he knows if he sees me he’ll drop it because he knows I’m going to tell him off… …Whereas if [female owner] was to walk into the room and he’s got my shoe, and she says “stop it” he’ll sort of run around, probably doing a round of the room you see, but once he sees me he’ll drop it, and he lays down as if to say “I’ve done something wrong, I shouldn’t of done that”. So, I’m just generally harder with him [than the female owner], you know, because I want him to be a well-behaved dog.*”

(Guardian of a young companion dog)

#### 3.2.3. Adaptations of Dog and Human to the Relationship and an Evolution of the HDB (Theme 3)

**Figure 1:**
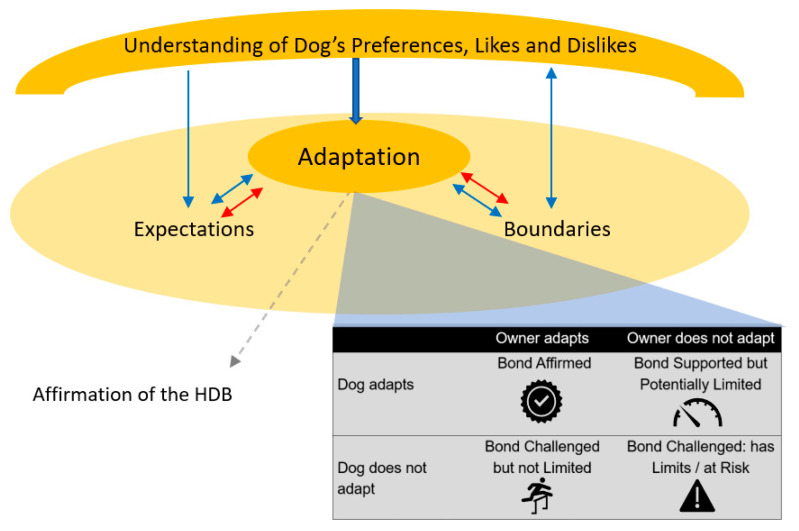
Suggested theory of how the qualitative themes, which emerged through semi-structured interviews, may relate to one another inside a human–dog bond (HDB). Solid lines propose there is a direct impact on the HDB, while the dotted line proposes a non-direct impact.

Within evolutionary theory, a key aspect to creating a unique and successful species is the ability of said species to adapt. If we consider the HDB as the unique entity between owner and dog, then the ability of the two parties to adapt to one another can perhaps predict its success (schematic example in Figure 1). Whether guardians receive, or need to receive, clear and regular affirmations from their dogs, whether guardians are understanding and/or are respectful of their dog’s preferences (and whether this latter aspect impacts the dog’s feelings towards their owner), all feed into the HDB. This will be affected by both parties’ previous experience, personality and temperament type, and expectations, but above all else, whether each individual can adapt to what the other offers in terms of characteristics around these factors. The final theme noted from the interviews was owner and dog adaptations to that unique relationship, which included both parties’ boundaries and expectations in and of the HDB. This quote from the guardian of an assistance dog highlights how these three elements are interconnected:

“*I think I just put some of the rules out of the window—with the assistance dog charity’s permission as well—and she was allowed on the bed and we didn’t worry about her pooing in a specific place and then we started to really bond, and we’ve [had] a really good bond since.*”

##### Expectations (Theme 3 Subtheme)

It is likely that humans have several conscious and unconscious expectations from a dog and an HDB in any relationship. This may relate to previous knowledge and/or experience of dogs, such as the example below:

“*I mean, I guess collies and German shepherds are traditionally kind of bonded dogs but again, I don’t know whether that was a breed related thing or whether that was just because of his background and the way he’d been kind of raised.*”

(Guardian of an older rehomed dog)

Humans may also have expectations they place on themselves as guardians. An expectation of the ease of training is one such example. A canine behaviourist describes a common misconception from dog guardians:

“*[many guardians believe that] this is brand new, but I should be Einsteining[sic] it by the end of this training session, which we know is not the case because they are individuals. And actually, it is understanding that we need to do a lot of work to buy the dog into what we’re doing, but then we can fade out the use of any tangible rewards and reinforcers if needs be.*”

(Dog guardian who also works with dogs)

Preconceptions about characteristics of the dog–human relationship could be seen as disappointing, or even a deal breaker in some cases, if guardians were not willing to accept the reality of the relationship. It would be difficult to measure whether a dog’s expectations are met in a relationship; however, it could be argued that it can be measured if a dog’s expectations are exceeded by signs of an improvement in trust of a guardian/s over time:

“*I think when we first got her, he [the partner] was very much hoping that she [the dog] would be if you like “his” dog, as historically quite a few [of our dogs] have kind of gravitated to me, mainly because they had more time with me because of our working pattern. So originally, I think he was anticipating, or hoping, that she would be his dog, more than my dog, but actually as it turned out, she was very, very, wary of us both—but him particularly—just being a man, so it did take him longer, him and her longer, to kind of figure each other out and decide what they like and what they don’t like, but now I would say she’s fully equally bonded with both of us, just in slightly different ways.*”

(Guardian of a rehomed dog)

Letting go of expectations, arguably a form of adaptation to the reality of the HDB, appears to strengthen the bond between some humans and dogs, as this guardian of a working dog describes:

“*[Some days I think] “I’m going to let you rest, let you be a dog, let’s go for a walk”. And I don’t need to do any more with [her] or don’t want to do anything today. And actually, it’s probably made [our]—in terms of a working relationship—I’ve always had a really good, strong working relationship with [dogs] but probably the working relationship with [the dog] is even stronger [now] because when it does happen, it’s probably not as repetitive, as boring, or the pressures not [there].*”

(Guardian of an agility dog)

For dogs, expectations from relationships are likely to come from experience and the repetition and association of certain human’s care-giving roles within their life. This may be a positively reinforced expectation (e.g., feeding or walking if a dog enjoys or is motivated by these things) or a negatively reinforced association (e.g., veterinary visits if a dog dislikes medical procedures or car journeys). These different associations/expectations may form nuances in the type of HDB a dog shares with a human. Here, an interviewee described her dogs’ behaviours around different humans within their social network, who provided different resources to the dogs:

“*I would say that the one [bond] they had with my partner were very affectionate and they were quite happy to chill out with him. I would say that with my dog walker… …they tend to get a lot more excitable when she comes in the house. Rather than just “oh your home hiya”, they’re like “OH MY GOD! A walk!” And it’s kind of the same with my mum, when she comes over to visit, she’s really bad for feeding them treats—no matter how often I ask her not too! If mum’s there, they’ll sit at her feet. It’s a learned behaviour but they expect it so they’re a lot more excitable…*”

(Guardian of a rehomed dog who also works with dogs)

Expectation can also form trust from a dog; the expectation that someone will or will not hurt them can impact the HDB. Whether a dog trusts and is comfortable with a human can usually be read from a dog’s behaviour. The opposite is also true. Here, a woman describes the change to a bond she encountered with a dog she cared for:

Participant: “*We used to walk him every day, got on really well with him, he got really excited bouncing back in his kennel. We were working on doing a grooming programme with him because he had really bad matts in his ears, but he wasn’t great at handling, so we had weeks and weeks of giving him cuddles, introducing him to the type of scissors we were going to use, making the noise around his head, and then when it came to actually touching him with the scissors—not to cut, just to touch—the minute I touched his ears with the scissors he turned, ran at me, growling, barking, growling at me, and obviously I put the scissors away and everything calmed down. But after that I’d probably say for about a week, he wasn’t as excited to see me anymore, he walked to the back of the kennel when I came towards the kennel to get him out. We did get that bond back, but I would say that he was a lot more worried about me being around him because he thought I might have the scissors again.*”

Interviewer: “*That’s really interesting, and quite unusual circumstances so an interesting example. Do you think it took you longer afterwards to build that bond back up with him?*”

Participant: “*yes definitely, he would do a lot of body language, like, he would kind of give me side-eye… …so it did take him a long time to realise I wasn’t going to do it again but to trust me again I would say.*”

Interviewer: “*What behaviours did he show when you got that trust back?*”

Participant: “*He was more excitable and waggy in his kennel, excitable when I came in, he stopped showing me side-eye… a lot more relaxed.*”

(Guardian of multiple dogs who worked with dogs too)

This last quote is also an example of a boundary in the relationship set by the dog and could, therefore, also be included in the section below on boundaries.

##### Boundaries (Theme 3 Subtheme)

Boundaries link closely into the previous section’s theme of dog and human dislikes. Knowledge of one another’s dislikes may form boundaries in the HDB; however, if each party is respectful of them, they can build trust, a feeling of safety, and sometimes more dependence in a relationship. Often, it is the dog’s boundaries in a relationship that lead guardians to believe not everyone could be their owner.

Interviewer: “*so almost because she needs that little bit of extra understanding? And you have that, do you think that makes you feel closer?*”

Participant: “*definitely you almost have to have all eyes on her in certain scenarios… …not everyone could be her owner… …I think she would be a tricky dog. For some people in those scenarios, especially taking her to the vet, because of her pain she will react… …But, you know, it doesn’t faze me, and I know that the pain is there, but I try and make [it] as pain free as possible with her.*”

(Guardian in a multiple-dog household)

“*[Dog] has had her own different tricky areas and in there as well which has taught me so many things, but it’s not taught me to decipher them, just like, oh, how do I cope with this?*”

(Guardian of an assistance dog)

Knowing and adapting to each other’s boundaries can assist the relationship, as this participant suggests:

“*I think what dogs like is rules and they want to know what they are allowed to do and what they’re not allowed to do, and so my partner moved in, and he didn’t want the dog to sleep on the bed with us. Fair enough it’s not a massive bed there’s not a load of room so suddenly the rule has changed for [dog] and she doesn’t get that, and she’s struggling as to why she now needs to sleep on the floor in her bed and not in my bed and I think that was the big problem, and so I had to compromise with my partner I said you know because you know he didn’t want the dog on the bed full stop. And I had to work with him to change that, you know not to change him but to say It’s actually really important for [dog] that she’s allowed on the bed sometimes and so every morning now if I’m not working [partner] leaves and [dog] gets on the bed and we have a cuddle—and that’s a really important part of our week.*”

(Guardian of a single dog)

Management of boundaries can sometimes cause extra lifestyle adaptations and effort for the guardian:

“*[Dog] would not like unknown dogs around him so that meant in the house sometimes we had, not issues, because with his own group he was fine. But as [the dog] got older, basically when I first got [other dog] I couldn’t have [other dog] and [first dog] in the house together… …I had to do lots of management.*”

(Guardian of a multi-dog household)

How far an owner is willing to adapt to meet a dog’s need may be influenced by many environmental factors. The cost-to-benefit ratio of adaptation is worthy of exploration in future studies.

## 4. Discussion

The overall aim of this work was to explore the current limitations in lines of questioning around the HDB, and to propose potential themes for improvements to current tools for a more complete assessment of the HDB. The work here highlights how dog investment in the HDB is currently underrepresented in HDB tools. Yet, understanding dog investment in HDB can provide us with greater insights into how and why some relationships are successful while others fail, increasing relinquishment risk for the dog (and potentially the risk of euthanasia) [17,18]. As social mammals, a positive HDB can provide benefits to both parties and contribute to both the dog and human’s wellbeing [19,20,21], the latter typically being where most research is focused. As an adaptable species, dogs are regularly used for assistance, therapy, and training or teaching, and it is, therefore, important that we can measure their experience of the relationships in their lives to ensure that they gain as many of the benefits from these interactions as humans do.

A review of the literature (Part I) highlighted the imbalance between dog versus human investment questions in current HDB tools and, therefore, identified a potential flaw in construct design: not measuring both sides of the relationship equally. Therefore, it can be questioned whether the true definition of bond is actually being measured. This imbalance was noted previously in work by Payne et al. [10] and Horowitz [9], while misuse of HAI terms, formerly noted by Wilson and Netting [8], can also lead to a failure to measure a bond, instead commonly measuring human attachment. Payne et al. [10] pointed out that if the HDB is supposedly symbiotic, then affective benefits to the dog, through attachment or otherwise, should also be considered. In other words, failure to measure both parties’ interactions in the relationship is a failure to measure the bond.

Behavioural measurement of a dog’s investment in a relationship has been attempted using the Ainsworth Strange Situation Test (ASST). This test has been used to assess attachment styles of dogs, with some authors also using it to help validate human-centric tools (e.g., [22,23,24,25]). Behavioural validation is essential to ensure that anthropocentric evaluation of canine state accurately reflects the reality for dogs themselves. Yet, these tests alone may not capture all the nuances of a relationship, and use of other approaches in addition could allow for a wider perspective of the relationship, facilitating a more in depth understanding [23].

The themes that emerged from qualitative interviews are summarised in Figure 1. Adaptation by both dog and human to the HDB appears to be a cornerstone in determining the type of relationship said dog and human will have (as shown in the grey box). Boundaries and expectations within all aspects of the HDB may create limitations or opportunities within a relationship to improve its quality, as might an understanding of a dog’s preferences, likes, and dislikes. Whilst not necessary for a relationship, these factors, if understood and respected, could have an overarching impact on all aspects of the HDB and the distinct factors within it. Meanwhile, the “signs of affirmation”, which guardians perceive indicate bond existence, are not actually needed for an HDB but are reaffirming to the human. That is, reaffirming that the relationship, and the positive feelings about it, are mutual and that their investment in the relationship is being rewarded by return. The themes identified can facilitate future research exploring new dog-investment-based HDB questions using this foundation of preliminary work (such questions would require validity and reliability testing in tools). The themes that emerged here reflect how and why a dog might invest in the HDB and allow development of hypotheses of different bond types depending on both parties’ investment (as has been suggested within human–dog attachment [26]).

There are likely to be many other factors within the larger oval in Figure 1 (that encompasses adaptation, expectations, and boundaries) that influence each HDB in a unique way and could be explored further in future research. These other factors include training methods [27,28], individual differences or each party’s personality, previous experiences, aspects of the environment, other relationships and social support networks, husbandry responsibilities, time spent together, and quality of experiences shared. The three main themes that emerged in this study are discussed in more depth below.

### 4.1. HDB Affirmation

Perceived HDB affirmation behaviours cannot be assumed to be related to a dog’s investment in the bond but were recurrently reported in Part II of this study as important to humans within bonds. Nagasawa et al. (2008) presented physiological evidence that guardians can experience an increase in urinary oxytocin from their dog simply gazing at them [29]. Therefore, it is likely that perceived bond affirmation behaviours from dogs are physiologically very reinforcing to guardians. This has not gone unnoticed in current HDB assessment tools with the inclusion of questions such as “My dog gets excited when I come home” [30], and “My dog is constantly attentive to me” [31] in attempt to obtain a reading of how the dog might exhibit bond-affirming behaviours towards their humans. However, from a dog’s point of view, several emotional states could be associated with these contexts, e.g., excitement, fear, anxiety, or multiple conflicting states associated with previous learnt experience [32]. Alternatively, they may be related to previous positive reinforcement history, such as maintaining proximity to the human that feeds them. Human interpretation of affirmation behaviour does not necessarily indicate the type of emotions, relationship, or bond a dog has with a human; however, based upon these interviews, they are important to dog guardians in helping them gauge whether their dog is bonded to them.

Successful recall and obedience were commonly mentioned by dog guardians as indicative or affirming of a bond, although these responses will be influenced to a great extent by prior training and the dog’s interest in other aspects of an environment. Poor recall was often viewed by guardians as frustrating, embarrassing, or insulting. However, to the contrary, within parent–child secure-base attachment theory, children that were more likely to confidently explore the world away from the parent were considered to feel more secure in their bond [33], and similar secure-base effects have been noted between securely attached dogs and their guardians (e.g., [34]). This demonstrates what guardians perceive as indicative of a bond might not be so, and vice versa. It has been noted in past studies that guardians often have a limited understanding of the relationship between a dog’s emotions and behaviour [35,36,37,38,39,40]. Coupled with the possibility of anthropomorphism and the availability heuristic (i.e., using the most easily recalled information to provide context for decision-making processes [35,41,42]), HDB surveys may struggle to tease apart the dog’s true investment unless objectively relevant dog behaviour questions are included to assess HDB.

### 4.2. Understanding of Dogs’ Preferences, Likes, and Dislikes

An understanding and respect for a dog’s unique preferences, likes, and dislikes could be interpreted as an accurate knowledge of a dog’s behavioural motivators and reinforcers. These are key concepts to recognise when working with, training, and promoting a harmonious relationship with any animal; how this knowledge is utilised, respected, and understood could be the difference between simply repeated HAIs and forming a HDB.

Acknowledgement of dogs’ preferences can be found in some HDB questionnaires (e.g., “My dog usually plays by himself/herself or someone else instead of me, even when I’m around” [31]). However, dog-preference-related questions were scarce in contrast to guardian-preference questions in which “likes” were heavily represented (see Table S1, Supplementary Materials). This limited representation does not ensure that dog preferences are recognised in HDB tool question content.

It is likely that time, proximity, and shared experiences in a variety of situations together assist guardians in learning more about a dog and their responses, reactions, and preferences in a multitude of contexts. This phenomenon is known as behavioural or local synchronicity [43,44,45,46]. For learning to occur however, the human party would still need to observe, acknowledge, and understand the emotions behind a behaviour within a situation [36]. For example, interpreting a dog barking as being badly behaved rather than recognising that the dog may be experiencing fear, anxiety, or frustration, would be a direct barrier to the guardian responding appropriately. Attitudes to animals and empathy for a dog’s feelings are just as important and may be the reason why so many current HAI surveys address these factors.

A guardian’s knowledge of situations in which their dog may experience anxiety or fear can allow them to support their pet (e.g., comforting a dog that fears fireworks). In demonstration of this, one of the interview participants said that their dog sought proximity with them when feeling unwell, even referencing a time when the dog had entered into the shower with them when seeking comfort for a digestive upset. If a human can ease a dog’s negative emotional state by providing a source of positive emotion (e.g., safety, comfort, and companionship), then this is likely to impact how the dog feels about that human, their general wellbeing, and their emotional state around that human. Whether the guardian is a “unique resource” to the dog, i.e., whether they can offer the dog something emotionally, which no one else can, may also impact the importance of the relationship to that animal. Dogs’ emotions related to these factors are likely to create a different type of HDB than those of a dog that cannot rely on a guardian for emotional support but may still choose to sit near them when in a room together. Dogs’ choice for proximity is a common question in HDB tools, e.g., “My pet is constantly at my side” [47,48] and “I feel as if my dog often stays physically closer to another family member or a friend than me” [31]. However, this could be influenced by time of day, anxiety, and/or which resource that human may be associated with, rather than sign of a bond.

Numerous tools offer questions about the dog being a source of comfort to the human (e.g., “When upset or anxious I turn to my pet” [49]) while none appear to investigate the human as a source of comfort to the dog. This may be related to these questions being removed during tool design if question reliability was poor, or else it could be argued that questions exploring a dog’s choice of proximity to a human begin addressing this as a topic. Without HDB tools simultaneously asking about training methods, a dog’s preferences, and a human’s response to those preferences, alongside verification through behavioural trials, it is difficult to interpret how a dog may feel about the relationship with their guardian. For example, whilst questions such as “My dog does not look at me often” [31] and “My dog is constantly attentive to me” [31] were highlighted as indicative of a dog’s investment in an HDB (Table S1), a similar question “My dog always pays attention to me and obeys me right away” [31] was not included because this appears much more linked to a dog’s training history rather than emotional investment in an HDB (see [27,28]). Future questionnaires would benefit from exploring the use of training method-based questions, alongside trialling question themes based on where a dog seeks comfort.

### 4.3. Adaptation in the HDB

A key theme to emerge from thematic analysis was adaptation (Figure 1) by both dog and human to support and encourage a relationship to be established and maintained. Sub themes for this category were ‘boundaries’ and ‘expectations’, with all three themes fitting into the basic outline for prosocial behaviours (i.e., helping each other, obeying rules, conforming to socially acceptable behaviour, and cooperating with each other [26]). In their argument for the prosociality of dogs towards humans, Bräuer suggested that most dogs were motivated to please humans, but they often had problems understanding what was being asked of them [26]. Here, we suggest that the reverse could also be true; humans can also be motivated to please their dogs but can find difficulty in understanding them. Criteria for adaptation, which appeared during the interviews, included behavioural flexibility, forgiveness, gaining knowledge or understanding, empathy, respect, and not crossing boundaries, along with occasional acceptance that a dog may not be able to “invest” as much into a bond, or behave within a relationship, quite as the human party might like.

One example of beneficial adaptation for dog–human affiliation is behavioural synchronization [43,44,45,46]. Duranton and Gaunet (2016) defined behavioural synchronization as both parties doing the same thing, at the same time, in the same place [46]. Although it is a reasonably new area of research within canine cognitive science, questions relating to behavioural synchronization have made appearances in past HDB tools. Such questions were often based on sleep (e.g., “He/she is encouraged to sleep on my bed at night” [49]), rest (e.g., “My pet and I watch TV together frequently” [47,48]), play (e.g., “I enjoy playing with my dog”, “I play fetch with my dog often”, “My dog often is not interested in playing with me”, and “My dog usually plays by himself/herself or someone else instead of me, even when I’m around” [31]), and walking arrangements (e.g., “I take my pet along when I go jogging or walking” [49]). However, existing questions on synchronisation are limited in how they address the dog’s point of view regarding the time spent together, not necessarily revealing the quality of the time spent together (i.e., the level of enjoyment and or enthusiasm each party experiences during the activity). Many questions also fail to address active engagement and agency between the dyad when participating in a shared activity. For example, when walking a dog, it can be a very two-way process in which there is engagement between both parties and both the dog and human make choices about the route, or it can be very one sided, human-led, with little true choice in the behavioural synchronicity. Exceptions would be questions that specifically describe an activity that requires engagement (e.g., throwing a ball for a dog on a walk) or an active choice to participate in the same activity, such as sleeping in the same bed at the same time. Equally, forced behavioural synchronization (e.g., placing a restless dog in a crate to “rest” when the owner rests) is not necessarily an example of synchronization as there is no agency involved on behalf of the dog. Future HDB tools would benefit from including questions relating to true forms of behavioural synchronicity between dog and human, particularly those in which humans have adapted to a dog’s behaviours or preferences on how they spend their time.

In general, adaptation occurs in response to a change in environment, situation, or routine. Changes result in a stress response, and behavioural adaptation leads to a reduction in this stress. Adaptive responses to change may not always be rapid or successful and may result in prolonged stress responses. For an animal (human or otherwise) to adapt, an initial stressor or stimulus is required. As the name implies, a stressor may be stressful to said animal before it adapts; therefore, refusal, failure, or a long period of time taken to adapt may have knock-on effects on an animal and/or their relationships. It is known that stress leads to elevated glucocorticoids, which have been shown to reduce social tolerance [50,51]. Chronic stress can impact social interactions, and this could further disrupt the relationship or bond formation between dog and owner. Hence, the ability of owner and dog to adapt rapidly to changes in the nature and context of their relationship seems to be an important element in the development and maintenance of a positive bond. Adaptation (including boundary and expectation changes) can be promoted by the guardian’s knowledge of their dog’s preferences—ideally, guardians should be able to distinguish between when their dog has adapted to something and is comfortable, and when their dog is merely tolerating it.

Adaptation is not a new theme in qualitative studies on HDB (e.g., [35]), and it addresses whether an animal (human or otherwise) would or could modify their behaviours, routines, and/or own preferences to suit another’s preferences, or benefit a bond (i.e., whether an altruistic bond could be formed). However, adaptation appears to be underrepresented in current HDB tools; although, some questionnaires have tried to ascertain relationship limits (a form of boundary) e.g., “If a 3-month-old puppy or kitten was having problems with destructiveness I would get rid of it” [52]. Boundaries and expectations within all aspects of the HDB may create limitations or opportunities in adapting to each other during the creation or maintenance of a bond (see Figure 1). These are likely to be unique to each dyad depending on environment, personality type, attitudes towards animals, communication style (and success), previous experience, etc., highlighting the unique nature of many HDBs. uture questions on adaptation, whether based on life examples or fictional scenarios, could be incorporated into questionnaire themes on dogs’ preferences, likes, and dislikes.

### 4.4. Limitations

This paper identifies a lack of dog-centric questioning in current HDB tools and presents the results of a preliminary investigation that identifies potential themes to develop questions to address this gap in future. Any new questions arising from these themes would still require full validity and reliability testing within a questionnaire format to ensure they meet the criteria needed to report accurate information within a tool. Whilst current tools lack lines of questioning on dog investment within the HDB, it is unknown whether this has arisen from HDB tool authors exploring and removing questions that lack validity or reliability during testing. This study used qualitative methods and a diverse purposive sample was sought (rather than random sampling); however, the themes generated in this study could be limited or biased to the participants that took part.

## 5. Conclusions

This study highlights an area for improvement in current HDB tools: the need for further consideration and representation of canine investment in the HDB. If a bond is to signify a two-way shared relationship, then an understanding of the investment from both parties is required. While there is no shortage of HDB tools (e.g., [53,54,55,56,57,58,59,60,61,62,63,64,65]), there remains a lack of true interpretation of the bond from both parties and little discussion on the types of bonds that may exist outside of relative strength. Until a more rigorous examination of dog investment into the HDB is completed, the content of the questions used for measurement remains scarce and they remain unreliable in their ability to decipher the dog’s investment in a bond. Consequently, existing measurements must be utilised with caution [66,67]. Here, we suggested question themes that could be expanded on in future studies to address the deficit of questions assessing the canine investment in a bond. This paper is focussed on the HDB; however, future work could investigate similar trends of underrepresentation in other non-human species within HAI questionnaires.

Better representation of the animal’s investment within human–animal relationships could further our understanding of HAIs and increase our awareness of factors that threaten bonds for one or both parties. Equally, it could provide greater knowledge on distinct types of bonds and the factors that solidify or strengthen them (which could benefit those adopting/adopted dogs [68]), as well as the overall impact bonds have on either invested parties’ wellbeing. It is hoped that the more informed dog guardians are on their dogs’ needs in various types of HDB, the greater the number of interventions and support measures that can be put in place to avoid their break-down. This could reduce the risk of dog relinquishment and/or poor welfare outcomes that may be associated with relationships breaking down.

## Figures and Tables

**Table 1:** Previously published definitions for the human–animal bond (HAB).

Reference	HAB Definition
Purdue University College of Veterinary Medicine (2019)	“A dynamic relationship between people and animals in that each influences the psychological and physiological state of the other.” [3]
American Veterinary Medical Association (2019)	“A mutually beneficial and dynamic relationship between people and animals that is influenced by behaviours essential to the health and wellbeing of both.” [4]
Society for Companion Animal Studies (2019)	“A close relationship between people and animals.” [5]
Russow (2002)	“A reciprocal and persistent relationship between a human and an animal and their interactions must support the wellbeing of both parties.” [6]
Johnson et al. (1992)	“An emotional attachment between an owner and their pet.” [7]

**Table 2:** The suitability criteria that HAI tool questions had to meet for assessing the human–dog bond (HDB).

Criteria	Description
1	Objectively worded and not ambiguous, nor containing conjecture, nor anthropomorphism.
2	Applicable to an HDB, i.e., not referring to an HAI with another species in a way that could never be used for a dog (e.g., “My bird often speaks to me when I enter the room”).
3	Applicable to a relationship beyond an HAI (e.g., bond, attachment).
4	Applicable to all types of dog guardian but aimed at adults (18+ years).
5	Original-duplicated questions were removed (where identifiable the original source was retained).
6	Compatible with the Dogs Trust ^1^ ethos to promote high welfare standards in all aspects of dog care (e.g., questions mentioning hitting a dog were vetoed so as not to promote or normalise this behaviour as an acceptable response).

^1^ Dogs Trust is a UK-based animal welfare charity, specialising in the welfare and well-being of dogs. Registered Charity Numbers: 227523 and SC037843.

**Table 3:** Selected demographic characteristics of the 12 semi-structured interview participants. Characteristics overlapped so that the same people were sometimes representing multiple categories.

Characteristics of Participants	Number of Interviews Ascertained
Rehomed dog/s	6
Dog/s owned since puppy	5
Multiple dog home	5
Multiple person home	11
Children in home	2
Owned dog/s > 5 years	5
Dog has a role other than being a pet (e.g., assistance)	3
Works with dogs	4
Identifies as male	3

**Table 4:** Results of independent content categorisation by two researchers (LS and HVW) for 228 questions related to the human–dog bond (HDB). The 228 questions can be seen in Supplementary Materials, Table S1.

LS = 18 Content Categories	HVW = 21 Content Categories
Affection Closeness (proximity) Attitudes towards animals & pets Care External Representation Confidante Communication Relationship Emotional Support Feelings towards pet Feelings towards self with pet Neutral or negative experiences Miscellaneous Play Positive experiences and emotions Purpose/meaning/responsibility Separation Support/dependence	One-to-one engagement Attitudes/feelings towards animals Changes in dog behaviour Companionship value Daily Care Dog Investment Emotional benefit Emotional dependence Feelings towards a specific pet Food General affection/physical affection Proximity dog Proximity human Responsible dog ownership/guardianship Self-improvement General Concern for Welfare Lifestyle adaption Miscellaneous Social benefits Training/obedience Understanding

**Table 5:** Question content categories for the 228 HDB questions were labelled independently by two researchers then grouped into ten broad themes (the percentage of questions in each broad theme in shown). Each theme was then classified as either human- and/or dog-centric (the original 228 questions can be seen in Supplementary Materials, Table S1).

Broad Themes	Question Content Categories	Human- or Dog-Centric
One-to-one engagement (11%)	One-to-one engagement (HVW) Proximity human (HVW) General affection/physical affection (HVW) Affection (LS) Play (LS)	Human and Dog
Attitudes towards animals and pets (1%)	Attitudes/feelings towards animals (HVW) Attitudes towards animals & pets (LS)	Human
Care & welfare (8%)	Care (LS) Daily Care (HVW) Food (HVW) General Concern for Welfare (HWV) Separation (LS)	Human and Dog
Companionship value/Emotional benefit (48%)	Confidante (LS) Companionship value (HVW) Communication (LS) Emotional Support/benefit/dependence (LS/HVW) Feelings towards pet (LS) Feelings towards self with pet (LS) Positive experiences and emotions (LS) Neutral or negative experiences (LS) Support /dependence (LS) Purpose/meaning/responsibility (LS) Social benefits (HVW) Self-improvement (HVW)	Human
Dog investment (8%)	Proximity dog (HVW) Closeness (proximity) (LS) Dog Investment (HVW) Changes in dog behaviour (HVW)	Dog
Feelings towards “that” (specific) pet (2%)	Feelings towards a specific pet (HVW) Relationship (LS) External Representation (LS) Understanding (HVW)	Human
Lifestyle adaption/Time Investment (13%)	Lifestyle adaption (HVW) Neutral or negative experiences (LS) Positive experiences and emotions (LS)	Human
Miscellaneous (5%)	Misc. (LS/HWV)	Misc.
Ownership/Guardianship (2%)	Responsible dog ownership/guardianship (LS)	Human
Training/obedience (2%)	Training/obedience (HVW)	Human and Dog

## Data Availability

The data presented in this study are available on request from the corresponding authors. The data are not publicly available due to ethical approval of participant informed consent that included survey respondents being informed that we will remove all personally identifiable information before sharing data with universities and/or research institutions.

## Supplementary Materials

The following supporting information can be downloaded at: https://www.mdpi.com/article/10.3390/ani12070805/s1, Table S1: The 228 HAI questions identified as suitable to measure HDB in HAI tools published up to 1 January 2019. Table S2: Semi-structured interview questions.
