# Supplementary material for: Exploring and Developing the Questions Used to Measure the Human–Dog Bond: New and Existing Themes
Source: Animals (Basel). 2022 Mar 22;12(7):805. doi: 10.3390/ani12070805 (PMC8996840; doi:10.3390/ani12070805)
Supplement: Supplementary file 1 [file animals-12-00805-s001.zip › animals-1577184-Supplementary Table S1.pdf]

Supplementary Material

The 228 HAI questions identified by the two researchers (LS and HVW) as suitable to measure human-dog bond (HDB) in HAI tools published up to 01.01.2019 (identified via systematic literature reviews from Wilson & Netting (2012) and Samet et al. (in press)). Many tools shared related questions/content and many of the questions were used in multiple sources; those with similar or identical wording are included together with both references displayed. The recognised sources are cited (based on methodological information provided by authors in discussion of tool development). * = The 18 questions categorised by researchers (LS + HVW) that were behaviourally measurable relating to dog investment in the HDB.

Table S1. The 228 HAI questions identified as suitable to measure HDB in HAI tools published up to 01.01.2019. * Questions relating to dog investment in the HDB that were categorised as behaviourally measurable. Corresponding reference numbers can be found in paper.

Questions/Statements	Example Sources
A reward would be offered for their return	Archer & Ireland (2011) Dog Attachment Questionnaire (DAQ) [49]
After a hard day, I like to spend time with my dog	Schneider et al. (2010) Quality of Life and Human Animal Bond [30]
At home, I sit with my dog close to me	Schneider et al. (2010) Quality of Life and Human Animal Bond [30]
Being close to my pet is pleasant for me	Zilcha-Mano et al. (2011) Pet Attachment Questionnaire (PAQ) [67]
Do you keep a picture of your pet in your wallet or on display at your home or office?	Stallones et al. (1990) Quality of Attachment to Companion Animals [53]
Expresses ownership of pet	Melson (1998) Pet Attachment Scale – Revised (developed for children) [54]
Extra care is taken to ensure my dog does not escape or get lost	Archer & Ireland (2011) Dog Attachment Questionnaire (DAQ) [49]
Extra care is taken to ensure my dog is well taken care of whilst on holiday	Archer & Ireland (2011) Dog Attachment Questionnaire (DAQ) [49]
Gets angry or annoyed about pet	Melson (1998) Pet Attachment Scale – Revised (developed for children) [54]
Giving up my dog will be more difficult than any other dog I have raised in the past (if applicable)	Fratkin (2015) Relationship Questionnaire (RQ) [31]
Having a dog increased my self-esteem and self-worth	Archer & Ireland (2011) Dog Attachment Questionnaire (DAQ) [49]
Having a dog is a source of contact and comfort	Archer & Ireland (2011) Dog Attachment Questionnaire (DAQ) [49]
Having a dog means you cannot do what you want to	Archer & Ireland (2011) Dog Attachment Questionnaire (DAQ) [49]
Having a pet gives me something to care for	Zasloff (1996) Comfort from Companion Animals Scale (CCAS) [64]
Having a pet gives me something to love	Zasloff (1996) Comfort from Companion Animals Scale (CCAS) [64]
Having pets is a waste of money	Templer et al. (1981) Pet Attitude Scale (PAS) [55]
Having to deal with the death of my dog would be very hard	Archer & Ireland (2011) Dog Attachment Questionnaire (DAQ) [49]

He/she is encouraged to sleep on my bed at night	Archer & Ireland (2011) Dog Attachment Questionnaire (DAQ) [49]
House pet/s / my pet adds happiness to my life	Templer et al. (1981) Pet Attitude Scale (PAS) [55] / Geller (2005) Pet Attachment Scale (PATS) [65]
How hard is it to look after your dog	Dwyer et al. (2006) Monash Dog Owner Relationship Scale (MDORS) [62]
How often did you clean up after your companion animal?	Poresky et al. (1987) Companion Animal Bond Scale (CABS) [56]
How often did you hold, stroke or pet your companion animal?	Poresky et al. (1987) Companion Animal Bond Scale (CABS) [56]
How often did your companion animal sleep in your room?	Poresky et al. (1987) Companion Animal Bond Scale (CABS) [56]
How often do you tell your dog things you don't tell anyone else	Dwyer et al. (2006) Monash Dog Owner Relationship Scale (MDORS) [62]
How often does your dog stop you doing things you want to?	Dwyer et al. (2006) Monash Dog Owner Relationship Scale (MDORS) [62]
How often were you responsible for your companion animal's care?	Poresky et al. (1987) Companion Animal Bond Scale (CABS) [56]
I always let my dog sleep in the crate in my bedroom instead of some other place in the house	Fratkin (2015) Relationship Questionnaire (RQ) [31]
I am affected by the way others react to my pet	Cromer & Barlow (2013) Pet Attachment and Life Impact Scale (PALIS) [63]
I am emotionally dependent on my pet	Geller (2005) Pet Attachment Scale (PATS) [65]
I'm not very attached to my pet	Zilcha-Mano et al. (2011) Pet Attachment Questionnaire (PAQ) [67]
I am proud of my pet	Angle et al. (1993) Pet Bonding Scale (PBS) [57]
I am the one most likely to notice when my dog is not feeling well	Fratkin (2015) Relationship Questionnaire (RQ) [31]
I am the person that checks to make sure my dog has water on a daily basis	Fratkin (2015) Relationship Questionnaire (RQ) [31]
I am the person that feeds my dog on a daily basis	Fratkin (2015) Relationship Questionnaire (RQ) [31]
I believe my pet is my best friend	Holcomb et al. (1985) Pet Attachment Survey (PAS) [58]
I believe that pets should have the same rights and privileges as family members	Holcomb et al. (1985) Pet Attachment Survey (PAS) [58]
I can be myself with you	Davis & Juhasz (1995) Pet Friendship Scale (PFS) [59]
I confide in my pet	Geller (2005) Pet Attachment Scale (PATS) [65]
I consider my pet to be a friend	Holcomb et al. (1985) Pet Attachment Survey (PAS) [58]
I consider my pet to be a great companion	Holcomb et al. (1985) Pet Attachment Survey (PAS) [58]
I count on my pet being there when I need comfort	Geller (2005) Pet Attachment Scale (PATS) [65]
I do not feel like I have an emotional connection with my dog	Fratkin (2015) Relationship Questionnaire (RQ) [31]

I do not often call or email the puppy sitter to ask about my dog when I am away	Fratkin (2015) Relationship Questionnaire (RQ) [31]
I do not often talk about my dog to other people	Fratkin (2015) Relationship Questionnaire (RQ) [31]
I do not pet my dog frequently	Fratkin (2015) Relationship Questionnaire (RQ) [31]
I do not understand my dog very well	Fratkin (2015) Relationship Questionnaire (RQ) [31]
I don’t feel comfortable opening up to pets	Beck & Madresh (2008) Pet and Partnership Scale (PPS) [60]
I enjoy playing with my dog	Fratkin (2015) Relationship Questionnaire (RQ) [31]
I enjoy showing other people pictures of my pet	Holcomb et al. (1985) Pet Attachment Survey (PAS) [58]
I enjoy watching my pet	Zasloff (1996) Comfort from Companion Animals Scale (CCAS) [64]
I feel as if my dog is currently progressing well for his/her age	Fratkin (2015) Relationship Questionnaire (RQ) [31]
I feel distant from my pet	Zilcha-Mano et al. (2011) Pet Attachment Questionnaire (PAQ) [67]
I feel emotionally attached to my dog	Geller (2005) Pet Attachment Scale (PATS) [65] / Fratkin (2015) Relationship Questionnaire (RQ) [31]
I feel more relaxed in company when my dog is present	Archer & Ireland (2011) Dog Attachment Questionnaire (DAQ) [49]
I feel sad when I am separated from my pet	Geller (2005) Pet Attachment Scale (PATS) [65]
I feel satisfied with my relationship with my dog	Fratkin (2015) Relationship Questionnaire (RQ) [31]
I feel that my pet is a part of my family	Holcomb et al. (1985) Pet Attachment Survey (PAS) [58]
I find it easier to talk to my dog than to talk to people	Fratkin (2015) Relationship Questionnaire (RQ) [31]
I focus on my dog more when he/she is doing something wrong than when he/she is doing something right	Fratkin (2015) Relationship Questionnaire (RQ) [31]
I frequently talk to my pet	Templer et al. (1981) Pet Attitude Scale (PAS) [55] / Chumley et al. (1993) Military Companion Survey (MCS) [47]
I get comfort from touching my pet	Zasloff (1996) Comfort from Companion Animals Scale (CCAS) [64]
I get frustrated when my pet is not around as much as I would like it to be	Zilcha-Mano et al. (2011) Pet Attachment Questionnaire (PAQ) [67]
I give gifts to my pet for birthdays and special occasions	Chumley et al. (1993) Military Companion Survey (MCS) [47] / Kafer et al. (2002) Pet Relationship Scale (PRS) [48]
I go to you when I am lonely	Davis & Juhasz (1995) Pet Friendship Scale (PFS) [59]
I go to you when I am bored	Davis & Juhasz (1995) Pet Friendship Scale (PFS) [59]
I hate going home when my dog is not there to greet me	Archer & Ireland (2011) Dog Attachment Questionnaire (DAQ) [49]
I have a backup plan for managing my pet/s during a disaster if my first choice doesn't work	Trigg et al. (2017) Pet Owner Risk Propensity Scale (PORPS) [61]

I have attended most of the training classes for my dog	Fratkin (2015) Relationship Questionnaire (RQ) [31]
I have lots of fun with my pet	Angle et al. (1993) Pet Bonding Scale (PBS) [57]
I have no problem parting with my pet for a long duration	Zilcha-Mano et al. (2011) Pet Attachment Questionnaire (PAQ) [67]
I have regrets about getting my dog	Schneider et al. (2010) Quality of Life and Human Animal Bond [30]
I interact or have close contact with my dog for a majority of the time I am awake	Fratkin (2015) Relationship Questionnaire (RQ) [31]
I like my pet mostly because it is cute	Cromer & Barlow (2013) Pet Attachment and Life Impact Scale (PALIS) [63]
I like spending time with you	Davis & Juhasz (1995) Pet Friendship Scale (PFS) [59]
I like to cuddle with my pet	Cromer & Barlow (2013) Pet Attachment and Life Impact Scale (PALIS) [63]
I like to feed animals out of my hand	Templer et al. (1981) Pet Attitude Scale (PAS) [55]
I like to spend a lot of time with my pet	Angle et al. (1993) Pet Bonding Scale (PBS) [57]
I like to talk to my pet / about things that are important to me	Angle et al. (1993) Pet Bonding Scale (PBS) [57]
I like you the way you are	Davis & Juhasz (1995) Pet Friendship Scale (PFS) [59]
I look at my dog often	Fratkin (2015) Relationship Questionnaire (RQ) [31]
I love my pet	Geller (2005) Pet Attachment Scale (PATS) [65]
I miss my pet when I am away	Chumley et al. (1993) Military Companion Survey (MCS) [47] / Kafer et al. (2002) Pet Relationship Scale (PRS) [48]
I often find myself talking about my dog when in company	Archer & Ireland (2011) Dog Attachment Questionnaire (DAQ) [49]
I often incorporate play in to training sessions with my dog	Fratkin (2015) Relationship Questionnaire (RQ) [31]
I often show off my dog and talk about my dog's purpose to others	Fratkin (2015) Relationship Questionnaire (RQ) [31]
I participate in organized events with my dog	Schneider et al. (2010) Quality of Life and Human Animal Bond [30]
I play fetch with my dog often	Fratkin (2015) Relationship Questionnaire (RQ) [31]
I praise my dog when he/she performs well	Fratkin (2015) Relationship Questionnaire (RQ) [31]
I prefer to be with my pet more than others	Geller (2005) Pet Attachment Scale (PATS) [56]
I really like seeing pets enjoy their food / I like seeing my pet enjoy food	Templer et al. (1981) Pet Attitude Scale (PAS) [55]
I receive more companionship from friends or family than from my dog	Archer & Ireland (2011) Dog Attachment Questionnaire (DAQ) [49]
I regret getting a dog because of all the things I have to do to care for it	Schneider et al. (2010) Quality of Life and Human Animal Bond [30]
I regret getting my dog because of his/her behaviour problems	Schneider et al. (2010) Quality of Life and Human Animal Bond [30]
I share food with my pet	Chumley et al. (1993) Military Companion Survey (MCS) [47] / Kafer et al. (2002) Pet Relationship Scale (PRS) [48]
I sometimes give my dog table scraps	Fratkin (2015) Relationship Questionnaire (RQ) [31]

I spend a lot of time stroking and petting my dog	Archer & Ireland (2011) Dog Attachment Questionnaire (DAQ) [49]
I spend time every day training my dog	Fratkin (2015) Relationship Questionnaire (RQ) [31]
I spend time everyday playing with my pet	Chumley et al. (1993) Military Companion Survey (MCS) [47]
I take my pet along when I go jogging or walking	Kafer et al. (2002) Pet Relationship Scale (PRS) [48]
I take my pet with me to visit people	Cromer & Barlow (2013) Pet Attachment and Life Impact Scale (PALIS) [63]
I talk to my pet about things that bother me	Chumley et al. (1993) Military Companion Survey (MCS) [47] / Kafer et al. (2002) Pet Relationship Scale (PRS) [48]
I think about my dog frequently when we are not together	Fratkin (2015) Relationship Questionnaire (RQ) [31]
I think my pet is just a pet	Holcomb et al. (1985) Pet Attachment Survey (PAS) [58]
I think you are interesting	Davis & Juhasz (1995) Pet Friendship Scale (PFS) [59]
I think you are smart	Davis & Juhasz (1995) Pet Friendship Scale (PFS) [59]
I treat my dog as a dog, not as a person	Fratkin (2015) Relationship Questionnaire (RQ) [31]
I treat my pet to anything I happen to be eating if he/she seems interested	Chumley et al. (1993) Military Companion Survey (MCS) [47] / Kafer et al. (2002) Pet Relationship Scale (PRS) [48]
I walk my dog several times a day	Fratkin (2015) Relationship Questionnaire (RQ) [31]
I wish my dog and I never had to be apart	Dwyer et al. (2006) Monash Dog Owner Relationship Scale (MDORS) [62]
I wish my dog spent more time with someone else other than me	Fratkin (2015) Relationship Questionnaire (RQ) [31]
I worry about you	Davis & Juhasz (1995) Pet Friendship Scale (PFS) [59]
I would be very upset if something happened to my pet	Angle et al. (1993) Pet Bonding Scale (PBS) [57]
I would respond similarly to both potential harm to my pet/s and potential harm to a close person	Trigg et al. (2017) Pet Owner Risk Propensity Scale (PORPS) [61]
I would try to keep my animals as close to me as possible when responding to disaster threat	Trigg et al. (2017) Pet Owner Risk Propensity Scale (PORPS) [61]
I/we do not celebrate my dog's birthday	Archer & Ireland (2011) Dog Attachment Questionnaire (DAQ) [49]
If a 3-month-old puppy of kitten was having problems with destructiveness I would get rid of it	Staats et al. (1996) Miller-Rada Commitment to Pets Scale [52]
If a pet destroyed a $4,000.00 piece of furniture or personal item, I would get rid of it.	Staats et al. (1996) Miller-Rada Commitment to Pets Scale [52]
If a pet destroyed a $50.00 piece of furniture or personal item, I would get rid of it	Staats et al. (1996) Miller-Rada Commitment to Pets Scale [52]
If a young pet required extensive veterinary care, I would get rid of it.	Staats et al. (1996) Miller-Rada Commitment to Pets Scale [52]

If an adult dog or cat was having problems with destructiveness, I would get rid of it	Staats et al. (1996) Miller-Rada Commitment to Pets Scale [52]
If an adult dog or cat was having problems with housebreaking, I would get rid of it	Staats et al. (1996) Miller-Rada Commitment to Pets Scale [52]
If an emergency assembly area or shelter does not take my pet/s, I will not stay there without them, even if I feel it is my safest option	Trigg et al. (2017) Pet Owner Risk Propensity Scale (PORPS) [61]
If an old pet required extensive veterinary care, I would get rid of it.	Staats et al. (1996) Miller-Rada Commitment to Pets Scale [52]
If I am on holiday without my dog, I hardly ever think about him or her	Archer & Ireland (2011) Dog Attachment Questionnaire (DAQ) [49]
If I am separated from my pet/s and unaware of their status, it would become my foremost concern.	Trigg et al. (2017) Pet Owner Risk Propensity Scale (PORPS) [61]
If I can't get my pet to show interest in me, I get upset or angry	Zilcha-Mano et al. (2011) Pet Attachment Questionnaire (PAQ) [67]
If I have to evacuate under immediate threat, I would never delay departure on account of my pet/s	Trigg et al. (2017) Pet Owner Risk Propensity Scale (PORPS) [61]
If moving my pet/s at the absolute last minute would risk my safety I would rather leave them in place	Trigg et al. (2017) Pet Owner Risk Propensity Scale (PORPS) [61]
If necessary, I would be able to give away my pet without any difficulties	Zilcha-Mano et al. (2011) Pet Attachment Questionnaire (PAQ) [67]
In the event of a disaster, my most important concern is staying aware of my pet/s safety	Trigg et al. (2017) Pet Owner Risk Propensity Scale (PORPS) [61]
It bothers me that my dog stops me doing things I enjoyed doing before I owned it	Dwyer et al. (2006) Monash Dog Owner Relationship Scale (MDORS) [62]
It is annoying that I sometimes have to change my plans because of my dog	Dwyer et al. (2006) Monash Dog Owner Relationship Scale (MDORS) [62]
It makes me mad that I don't get the affection and support I need from my pet	Beck & Madresh (2008) Pet and Partnership Scale (PPS) [60]
It's worth giving up other things in life in order to have a pet	Cromer & Barlow (2013) Pet Attachment and Life Impact Scale (PALIS) [63]
It is easy for me to be affectionate with my pet	Beck & Madresh (2008) Pet and Partnership Scale (PPS) [60]
My dog always pays attention to me and obeys me right away	Fratkin (2015) Relationship Questionnaire (RQ) [31]
My dog and I go through the same basic routine every day	Fratkin (2015) Relationship Questionnaire (RQ) [31]
My dog and I spend quiet time such as watching TV, reading, or doing homework together	Fratkin (2015) Relationship Questionnaire (RQ) [31]

My dog chews on things that he/she is not supposed to	Fratkin (2015) Relationship Questionnaire (RQ) [31]
My dog costs too much money	Dwyer et al. (2006) Monash Dog Owner Relationship Scale (MDORS) [62]
My dog does not always respond when I give him/her commands	Fratkin (2015) Relationship Questionnaire (RQ) [31]
My dog does not comfort me when I am upset	Fratkin (2015) Relationship Questionnaire (RQ) [31]
*My dog does not follow me around the house very often	Fratkin (2015) Relationship Questionnaire (RQ) [31]
*My dog does not look at me often	Fratkin (2015) Relationship Questionnaire (RQ) [31]
My dog does not stay on command	Fratkin (2015) Relationship Questionnaire (RQ) [31]
*My dog follows me wherever I go	Fratkin (2015) Relationship Questionnaire (RQ) [31]
*My dog gets excited when I come home	Schneider et al. (2010) Quality of Life and Human Animal Bond [30]
My dog has improved my mental health	Schneider et al. (2010) Quality of Life and Human Animal Bond [30]
My dog helps me get through tough times	Dwyer et al. (2006) Monash Dog Owner Relationship Scale (MDORS) [62]
My dog helps reduce (my) anxiety	Schneider et al. (2010) Quality of Life and Human Animal Bond [30]
My dog helps reduce (my) stress	Schneider et al. (2010) Quality of Life and Human Animal Bond [30]
*My dog initiates play with me several times a day	Fratkin (2015) Relationship Questionnaire (RQ) [31]
My dog is an important part of my life	Archer & Ireland (2011) Dog Attachment Questionnaire (DAQ) [49]
*My dog is constantly attentive to me	Fratkin (2015) Relationship Questionnaire (RQ) [31]
My dog is left alone without people for several hours a day	Fratkin (2015) Relationship Questionnaire (RQ) [31]
My dog is there whenever I need to be comforted	Dwyer et al. (2006) Monash Dog Owner Relationship Scale (MDORS) [62]
My dog makes me feel good about myself	Schneider et al. (2010) Quality of Life and Human Animal Bond [30]
(I feel like) My dog makes too much mess	Fratkin (2015) Relationship Questionnaire (RQ) [31] / Dwyer et al. (2006) Monash Dog Owner Relationship Scale (MDORS) [62]
My dog often does not come right away when I call his/her name	Fratkin (2015) Relationship Questionnaire (RQ) [31]
*My dog often is not interested in playing with me	Fratkin (2015) Relationship Questionnaire (RQ) [31]
My dog often shows signs of distress (e.g., whining) when I am away	Fratkin (2015) Relationship Questionnaire (RQ) [31]
*My dog pays more attention to strangers than he/she does with me	Fratkin (2015) Relationship Questionnaire (RQ) [31]
*My dog shows more interest in me than in my family/friends	Fratkin (2015) Relationship Questionnaire (RQ) [31]
*My dog spends more time with me than he/she does with anyone else	Fratkin (2015) Relationship Questionnaire (RQ) [31]

*My dog usually plays by himself/herself or someone else instead of me, even when I'm around	Fratkin (2015) Relationship Questionnaire (RQ) [31]
*My dog usually walks away when I pet him/her	Fratkin (2015) Relationship Questionnaire (RQ) [31]
*My dog will come and sit next to me	Schneider et al. (2010) Quality of Life and Human Animal Bond [30]
*My dog will follow me around the house	Schneider et al. (2010) Quality of Life and Human Animal Bond [30]
My pet and I watch TV together frequently	Chumley et al. (1993) Military Companion Survey (MCS) [47] / Kafer et al. (2002) Pet Relationship Scale (PRS) [48]
My pet calms me down	Cromer & Barlow (2013) Pet Attachment and Life Impact Scale (PALIS) [63]
My pet cheers me up	Cromer & Barlow (2013) Pet Attachment and Life Impact Scale (PALIS) [63]
My pet gives me a reason for getting up in the morning	Chumley et al. (1993) Military Companion Survey (MCS) [47] / Kafer et al. (2002) Pet Relationship Scale (PRS) [48]
My pet gives me something that I can form a close emotional bond with	Cromer & Barlow (2013) Pet Attachment and Life Impact Scale (PALIS) [63]
My pet goes to the veterinarian for regular checkups and shots	Kafer et al. (2002) Pet Relationship Scale (PRS) [48]
My pet has feelings	Angle et al. (1993) Pet Bonding Scale (PBS) [57]
My pet helps me to be more physically active	Kafer et al. (2002) Pet Relationship Scale (PRS) [48]
My pet is a financial hardship	Cromer & Barlow (2013) Pet Attachment and Life Impact Scale (PALIS) [63]
My pet is a source of constancy in my life	Zasloff (1996) Comfort from Companion Animals Scale (CCAS) [64]
My pet is a valuable possession	Chumley et al. (1993) Military Companion Survey (MCS) [47] / Kafer et al. (2002) Pet Relationship Scale (PRS) [48]
My pet is constantly at my side	Chumley et al. (1993) Military Companion Survey (MCS) [47] / Kafer et al. (2002) Pet Relationship Scale (PRS) [48]
My pet is fun and entertaining	Cromer & Barlow (2013) Pet Attachment and Life Impact Scale (PALIS) [63]
My pet is my companion	Cromer & Barlow (2013) Pet Attachment and Life Impact Scale (PALIS) [63]
My pet makes me feel confident	Beck & Madresh (2008) Pet and Partnership Scale (PPS) [60]
My pet makes me feel important	Angle et al. (1993) Pet Bonding Scale (PBS) [57]
My pet makes me feel needed	Zasloff (1996) Comfort from Companion Animals Scale (CCAS) [64]
My pet means more to me than any of my friends	Holcomb et al. (1985) Pet Attachment Survey (PAS) [58] / Templer et al. (1981) Pet Attitude Scale (PAS) [55] / Geller (2005) Pet Attachment Scale (PATS) [65]
My pet provides me with pleasurable activity	Zasloff (1996) Comfort from Companion Animals Scale (CCAS) [64]
My pet provides stability for me	Cromer & Barlow (2013) Pet Attachment and Life Impact Scale (PALIS) [63]

*My pet stays close to me when I am upset	Angle et al. (1993) Pet Bonding Scale (PBS) [57]
My pet teaches me responsibility	Cromer & Barlow (2013) Pet Attachment and Life Impact Scale (PALIS) [63]
My pet teaches me to be more loving	Cromer & Barlow (2013) Attachment and Life Impact Scale (PALIS) [58]
My pet teaches me to trust	Cromer & Barlow (2013) Pet Attachment and Life Impact Scale (PALIS) [63]
One of my favourite things to do is spend time with my pet	Angle et al. (1993) Pet Bonding Scale (PBS) [57]
Owning a dog has improved my social life	Schneider et al. (2010) Quality of Life and Human Animal Bond [30]
Owning a dog helps me meet people	Schneider et al. (2010) Quality of Life and Human Animal Bond [30]
People are more important to me than my dog is	Archer & Ireland (2011) Dog Attachment Questionnaire (DAQ) [49]
Pet comes on family trips	Melson (1998) Pet Attachment Scale – Revised (developed for children) [54]
Pets take a lot of time but it is worth it	Cromer & Barlow (2013) Pet Attachment and Life Impact Scale (PALIS) [63]
Signs of affection from my pet bolster my self-worth	Zilcha-Mano et al. (2011) Pet Attachment Questionnaire (PAQ) [67]
Someone else other than me is usually the one that takes care of my dog	Fratkin (2015) Relationship Questionnaire (RQ) [31]
Sometimes my only friend is my pet	Angle et al. (1993) Pet Bonding Scale (PBS) [57]
Taking care of my dog has increased the stress in my life	Fratkin (2015) Relationship Questionnaire (RQ) [31]
Talking to my pet makes me feel better	Cromer & Barlow (2013) Pet Attachment and Life Impact Scale (PALIS) [63]
The loss of my dog would mean as much to me as the loss of a family member or friend	Archer & Ireland (2011) Dog Attachment Questionnaire (DAQ) [49]
There are major aspects of owning a dog I don't like	Dwyer et al. (2006) Monash Dog Owner Relationship Scale (MDORS) [62]
There are times I'd be lonely except for my pet	Chumley et al. (1993) Military Companion Survey (MCS) [47]
Thinking about my dog makes me feel good	Schneider et al. (2010) Quality of Life and Human Animal Bond [30]
Walking my dog relieves my stress	Schneider et al. (2010) Quality of Life and Human Animal Bond [30]
What I like about my dog is its acceptance, love and loyalty	Archer & Ireland (2011) Dog Attachment Questionnaire (DAQ) [49]
When I feel bad, I seek my pet for comfort	Geller (2005) Pet Attachment Scale (PATS) [65]
When I hear about extreme examples of people risking their safety to rescue their animals, I empathise and feel I would likely do the same thing	Trigg et al. (2017) Pet Owner Risk Propensity Scale (PORPS) [61]

When I'm alone, I often think about my dog	Archer & Ireland (2011) Dog Attachment Questionnaire (DAQ) [49]
When I'm away from my pet for a long period of time, I hardly think about it	Zilcha-Mano et al. (2011) Pet Attachment Questionnaire (PAQ) [67]
When my dog is upset, I give him/her time to return to a calm emotional state	Fratkin (2015) Relationship Questionnaire (RQ) [31]
When people let me down I don't find that I rely more upon my dog for companionship and solace	Archer & Ireland (2011) Dog Attachment Questionnaire (DAQ) [49]
When talking to my dog I often use endearing terms or baby talk	Archer & Ireland (2011) Dog Attachment Questionnaire (DAQ) [49]
*When you come home, your pet is the first one to greet you	Holcomb et al. (1985) Pet Attachment Survey (PAS) [58]
Without acts of affection from my pet I feel worthless	Zilcha-Mano et al. (2011) Pet Attachment Questionnaire (PAQ) [67]
You are too busy to spend time with your pet	Holcomb et al. (1985) Pet Attachment Survey (PAS) [58]
You buy presents for your pet	Holcomb et al. (1985) Pet Attachment Survey (PAS) [58]
You feel sad when you are seperated from your pet	Holcomb et al. (1985) Pet Attachment Survey (PAS) [58]
You ignore your pet when he/she approaches	Holcomb et al. (1985) Pet Attachment Survey (PAS) [58]
You like to touch and stroke your pet	Holcomb et al. (1985) Pet Attachment Survey (PAS) [58]
You show photos of your pet to your friends	Holcomb et al. (1985) Pet Attachment Survey (PAS) [58]
You spend time each day grooming your pet	Holcomb et al. (1985) Pet Attachment Survey (PAS) [58]
You talk to your pet as a friend	Holcomb et al. (1985) Pet Attachment Survey (PAS) [58]
*Your pet is aware of your different moods	Holcomb et al. (1985) Pet Attachment Survey (PAS) [58]
*Your pet tries to stay near by following you	Holcomb et al. (1985) Pet Attachment Survey (PAS) [58]
