# Supplementary material for: Exploring and Developing the Questions Used to Measure the Human–Dog Bond: New and Existing Themes
Source: Animals (Basel). 2022 Mar 22;12(7):805. doi: 10.3390/ani12070805 (PMC8996840; doi:10.3390/ani12070805)
Supplement: Supplementary file 1 [file animals-12-00805-s001.zip › animals-1577184-Supplementary Table S2.pdf]

Supplementary Material

Table S2. Semi-structured interview questions. Interviews began by reading out the definition for the HDB that participants had already been provided with in writing before the interview (i.e., "*a unique, dynamic and two-way (reciprocated) relationship between a person and a dog, one in which each member can influence the other's psychological and physiological state*").

**Semi-structured interview questions**
What are your thoughts on the definition of the HAB on the worksheet?
Using this definition can you tell me about the bond that you and your dog share?
In your experience, which behaviours tell you that your dog is bonded with you? (Prompt examples: what is it about that behaviour that makes you feel your dog is bonded to you? Are there certain situations in which this tends to occur?)
Can you describe a time or times when this bond felt or feels strongest? (Do you think your dog would agree with this or is there another time they may feel most bonded to you?)
Is there anyone else that shares a bond with your dog? (Is the bond between them different to the one you share and if so, how?)
Do you have or have you had other dogs or animals in your life that you've shared a different type of bond with, and can you tell me a bit about how that bond differed?
Can you give some examples of how your dog shows you that they are bonded with you?
Can you tell me about a time when the bond you share has ever been broken or has ever come under risk of being broken?
How do you think a dog might inform you that they are no longer bonded with you, or the bond has changed?
Do you think different type of dog-human bonds exist and how might you categorise them?
Is there anything else you feel that we should have talked about but didn't – related to sharing a bond with a dog?
Have we missed anything that you think is important?
